# Distinct Adipocyte Responses to Δ^9^-Tetrahydrocannabinol (THC) Exposure Govern Hepatic Lipid Accumulation in an Obesogenic Setting

**DOI:** 10.3390/ijms26188860

**Published:** 2025-09-11

**Authors:** Adi Eitan, Ofer Gover, Betty Schwartz

**Affiliations:** The Institute of Biochemistry, Food Science and Nutrition, The Robert H. Smith Faculty of Agriculture, Food and Environment, The Hebrew University of Jerusalem, Rehovot 9190401, Israel; adi.levy@mail.huji.ac.il (A.E.); ofer.gover@mail.huji.ac.il (O.G.)

**Keywords:** obesity, tetrahydrocannabinol (THC), adipocytes, hepatocytes, FFA-enriched conditions

## Abstract

The effects of Δ^9^-tetrahydrocannabinol (THC) on adipocyte function under obesogenic, free-fatty-acid (FFA)-rich conditions remain poorly characterized, particularly regarding adipogenesis, FFA buffering, and downstream hepatocyte lipid handling. We investigated THC’s effect on adipogenic differentiation, temporal FFA buffering in mature adipocytes under lipid stress, and hepatocyte lipid accumulation driven by extracellular FFAs. The 3T3-L1 preadipocytes were differentiated in 0.5 mM oleate: palmitate (2:1) medium with vehicle (EtOH), THC (1 μM), or rosiglitazone (30 μM). Adipogenesis was assessed using BODIPY/NucSpot 650 staining followed by lipid droplet (LD) analysis. Adipocytes (days 10–18) were monitored for lipid accumulation, LD morphology, lipolysis, extracellular non-esterified fatty acids (NEFA), and lipid-handling gene expression. Conditioned media (CM) were applied to AML12 hepatocytes to assess lipid uptake. By day 6, THC enhanced adipogenesis, increasing lipid accumulation. In mature adipocytes, THC induced a biphasic buffering response: on day 10, NEFA levels were elevated despite unchanged lipid content, with increased isoproterenol-stimulated lipolysis. By day 18, buffering improved, with enhanced lipid storage, elevated stimulated lipolysis, smaller LDs, and altered gene expression. AML12 lipid accumulation corresponded with residual NEFA in CM, indicating that adipocyte FFA sequestration modulates hepatocyte lipid uptake. These findings reveal that under FFA-rich conditions, THC promotes late-stage adipogenesis and remodels adipocyte lipid handling, regulating extracellular FFA availability and hepatocyte lipid loading.

## 1. Introduction

Obesity is a multifactorial condition marked by metabolic dysfunction, such as insulin resistance, ectopic lipid deposition, and low-grade inflammation, promoting metabolic disorders [1]. However, not all obese individuals develop liver pathology associated with metabolic dysfunction, previously termed non-alcoholic fatty liver disease (NAFLD) and now referred to as metabolic dysfunction-associated steatotic liver disease (MASLD). These metabolically healthy obese individuals are protected by efficient fat storage and healthy expandable adipose tissue [1,2].

Adipose tissue expands via (i) recruitment and differentiation of progenitor cells (hyperplasia) and (ii) hypertrophy of mature adipocytes [3]. The balance between these two processes impacts metabolic health; hypertrophic adipocytes exhibit impaired lipid buffering, hypoxia, and pro-inflammatory cytokines secretion, promoting insulin resistance and metabolic syndrome [4]. Conversely, a higher proportion of smaller adipocytes, reflecting active adipogenesis, correlates with better metabolic profiles, by efficiently sequestering triglycerides and limiting peripheral lipotoxicity [5,6].

Adipogenesis is a two-step process whereby fibroblast-like progenitor cells restrict themselves to the adipocyte lineage, forming preadipocytes, followed by differentiation, during which preadipocytes undergo growth arrest, accumulate lipids, and form insulin-responsive mature adipocytes [3]. The master regulator of adipogenesis is peroxisome proliferator-activated receptor-γ (PPARγ) [7]. Numerous lipid metabolites are proposed as endogenous ligands of PPARγ, including polyunsaturated fatty acids, eicosanoids, and prostaglandins [3]. Thiazolidinediones (TZDs), potent agonists of PPARγ, stimulate adipogenesis in vitro and in vivo and act as insulin sensitizers [8,9]. TZDs, such as rosiglitazone (ROSI), have shown promising therapeutic effects in MASLD patients by promoting fatty acid storage in adipose tissue; however, their clinical use is limited due to several adverse events [8].

Our previous in vivo work revealed that administration of Δ^9^-tetrahydrocannabinol (THC), the main psychotropic constituent of cannabis, improved glucose tolerance and ameliorated fatty liver parameters in obese rodents. These therapeutic effects were associated with reduced adipocyte size but unchanged epididymal fat weight [10]. Together, these findings suggest that THC may directly affect adipocyte lipid dynamics and thereby influence hepatic lipid accumulation, providing the rationale to examine its effects on adipocyte lipid handling under obesogenic conditions.

The present study explored how THC influences adipogenesis of 3T3-L1 murine preadipocytes under an obesogenic environment created by the exposure to free fatty acids (FFA; oleate/palmitate, 2:1), simulating the chronic lipotoxic milieu that adipocytes experience in obese individuals [11]. We systematically evaluated lipid accumulation, lipid droplet (LD) distribution, lipolytic activity, and extracellular non-esterified fatty acids (NEFA) at days 10, 14, and 18 of differentiation, with ROSI serving as a positive control. This study aimed to characterize how THC modulates adipocyte lipid handling and FFA buffering over time and to assess the potential downstream effects on hepatocyte lipid accumulation using conditioned medium (CM) experiments. Understanding these temporal effects may provide insight into how THC influences adipocyte phenotype and its potential role in obesity-associated metabolic dysfunction.

## 2. Results

### 2.1. Proliferation and Differentiation Efficacy of THC Compared to ROSI

**Figure 1:**
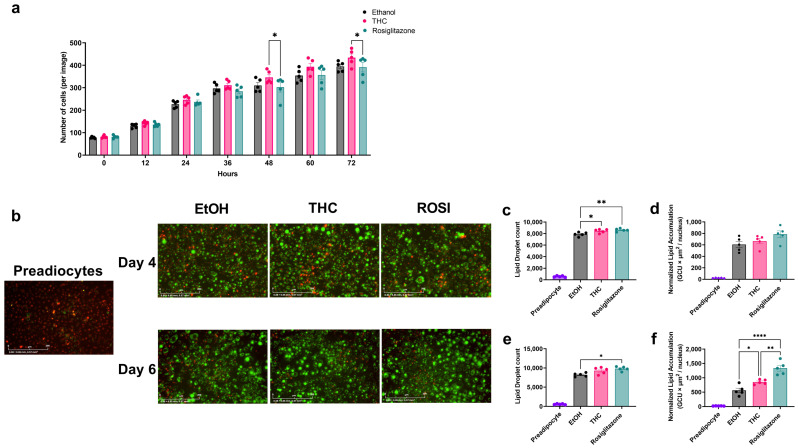
Effects of THC and ROSI on 3T3-L1 preadipocyte proliferation and differentiation under FFA-enriched conditions. (**a**) Preadipocyte proliferation over 72 h was monitored using the IncuCyte live-cell imaging system. Cell counts per image are shown for THC-treated (1 µM), ROSI-treated (30 µM), and vehicle (EtOH, 0.06%) control groups. (**b**) Representative fluorescence images of cells at days 4 and 6 of differentiation, stained with NucSpot650 (nuclei, red) and BODIPY (LD, green). Scale bar: 200 µm. (**c**) LD count per image on day 4 of differentiation. (**d**) Normalized lipid accumulation on day 4, calculated as integrated BODIPY fluorescence intensity divided by nuclear count. (**e**) LD count per image on day 6. (**f**) Normalized lipid accumulation on day 6. Data represent mean ± SEM (n = 5). Two-way ANOVA determined statistical significance with Tukey’s post hoc test for panel (**a**), and one-way ANOVA with Tukey’s post hoc test for panels (**c**–**f**). * *p* < 0.05; ** *p* < 0.01; **** *p* < 0.0001.

Preliminary dose–response experiments under obesogenic conditions identified 1 µM THC as the most effective non-cytotoxic concentration for promoting adipogenesis (Supplementary Figures S1–S3). To assess THC’s adipogenic capacity, we compared its effects on preadipocyte proliferation and differentiation to the PPARγ agonist ROSI. Time-lapse live-cell imaging revealed that THC induced greater proliferation than ROSI at 48–72 h post-treatment (Figure 1a). Differentiation was assessed via dual staining with NucSpot650 (nuclei) and BODIPY (neutral lipids) (Figure 1b). On day 4 of differentiation, both treatments increased LD number relative to vehicle, but normalized lipid content remained unchanged (Figure 1c,d). By day 6, both THC and ROSI treatments increased lipid content (Figure 1f). A PPARγ reporter assay confirmed that THC functions as a partial agonist, supporting its adipogenic effect (Supplementary Figure S4).

### 2.2. THC Promotes a Metabolically Favorable LD Distribution Pattern

**Figure 2:**
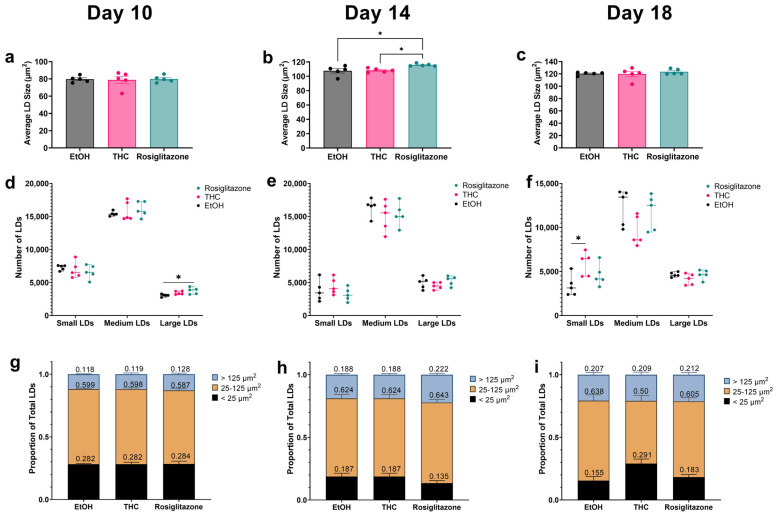
Effects of THC and ROSI on lipid droplet size dynamics in mature adipocytes. 3T3-L1 preadipocytes were differentiated and treated with vehicle (EtOH, 0.06%), THC (1 µM), or ROSI (30 µM) under FFA-enriched conditions, and LD size characteristics were analyzed at days 10, 14, and 18 post-induction of differentiation. (**a**–**c**) Quantification of average LD size per image for each treatment group at the respective time points, based on BODIPY staining and IncuCyte image analysis. (**d**–**f**) LD size frequency distribution per time point. LDs were classified into three size categories based on percentile calculations: small (<25 μm^2^), medium (25–125 μm^2^), and large (>125 μm^2^), as described in the Methods section. (**g**–**i**) Proportion of LDs in each size category for each treatment group at the respective time points. Data represent mean ± SEM from (n = 5). Statistical analysis: one-way ANOVA with Tukey’s post hoc test was used for comparing LD frequency within each size category (**d**–**f**); two-way ANOVA was used to assess the effects of treatment and LD size on proportional distribution (**g**–**i**). * *p* < 0.05.

To assess functional lipid storage, we tracked LD dynamics from days 10–16 of differentiation under FFA-rich conditions using BODIPY staining. Although both THC and ROSI enhanced lipid accumulation, they drove distinct LD remodeling patterns: ROSI progressively increased LD size (notably from day 14), whereas THC primarily increased LD numbers compared to ROSI (Supplementary Figure S5). To further examine these patterns at key time points, we performed morphometric analysis on days 10, 14, and 18. LD size did not differ between groups on day 10 (Figure 2a), but was significantly greater in ROSI-treated cells by day 14 compared to THC and vehicle (Figure 2b), with differences resolved by day 18 (Figure 2c). To capture treatment-specific changes in LD morphology, we categorized LDs as small (<25 μm^2^), medium (25–125 μm^2^), or large (>125 μm^2^), as detailed in the Methods. ROSI increased large LDs at day 10 (Figure 2d), but no significant differences were detected at day 14 (Figure 2e). Concomitantly, no significant differences in the distribution of LD size categories were detected at day 10 or 14 (Figure 2g,h). By day 18, THC increased the total number of small LDs compared to vehicle (Figure 2f). This was accompanied by a higher proportion of small LDs (29% vs. 15.5% in EtOH), while the proportion in ROSI-treated cells (18.2%) was not significantly different from THC (Figure 2i). Concurrently, the percentage of medium-sized LDs was reduced in THC-treated cells (50%) compared to EtOH controls (63.6%).

### 2.3. Assessment of THC’s Effect on Adipocyte Lipid Handling and FFA Buffering Capacity

**Figure 3:**
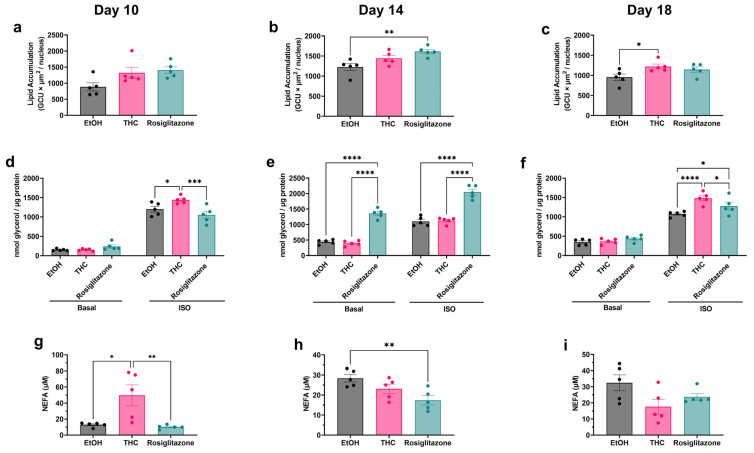
Effects of THC and ROSI on lipid handling in mature adipocytes. 3T3-L1 preadipocytes were differentiated and treated with vehicle (EtOH, 0.06%), THC (1 µM), or ROSIe (30 µM) under FFA-enriched conditions. Lipid handling parameters, including storage, mobilization (basal and ISO-stimulated lipolysis), and extracellular NEFA levels, were assessed at days 10, 14, and 18 post-induction of differentiation. (**a**–**c**) Normalized lipid accumulation at the respective time points was calculated by dividing the integrated BODIPY fluorescence intensity (neutral lipid signal) by the number of nuclei identified using NucSpot650. (**d**–**f**) Lipolysis was measured by glycerol release under basal and ISO-stimulated conditions at the represented time points, normalized to protein. (**g**–**i**) NEFA concentration in the culture medium at the represented time points. Data represent mean ± SEM (n = 5). Statistical significance was determined by one-way ANOVA followed by Tukey’s post hoc test for lipid accumulation and NEFA data; lipolysis was analyzed using two-way ANOVA. * *p* < 0.05, ** *p* < 0.01, *** *p* < 0.001, **** *p* < 0.0001.

Next, we assessed the temporal effects of THC on adipocyte FFA buffering by measuring normalized lipid accumulation, lipolysis, and medium NEFA. Lipid content was comparable across groups on day 10, though ROSI trended higher (*p* = 0.052) (Figure 3a). By day 14, ROSI significantly enhanced lipid storage, with THC showing an intermediate effect (Figure 3b). On day 18, THC significantly increased lipid accumulation relative to vehicle (Figure 3c). These trends differ from the non-normalized integrated intensity data (days 10–16, Supplementary Figure S5), which showed earlier increases for both treatments, highlighting the importance of normalization (see Methods). Lipolysis analysis revealed distinct temporal responses: THC selectively increased isoproterenol-stimulated lipolysis at day 10 (Figure 3d), whereas ROSI enhanced both basal and stimulated lipolysis at day 14 (Figure 3e). By day 18, both treatments augmented stimulated lipolysis (Figure 3f). NEFA measurements, reflecting net lipid flux amid exogenous FFA supplementation, were elevated in THC-treated cells at day 10 compared to vehicle and ROSI groups, despite similar basal lipolysis and non-significant differences in lipid accumulation (Figure 3g). On day 14, ROSI-treated cells exhibited lower NEFA levels than vehicle controls, despite increased lipolysis (Figure 3h). By day 18, NEFA levels were lower in THC and ROSI groups versus vehicle; THC showed a trend toward significance (*p* = 0.058) (Figure 3i).

### 2.4. Gene Expression Analysis Reveals Differential Regulation of Adipogenic and Lipid Metabolism Pathways by THC and ROSI

**Figure 4:**
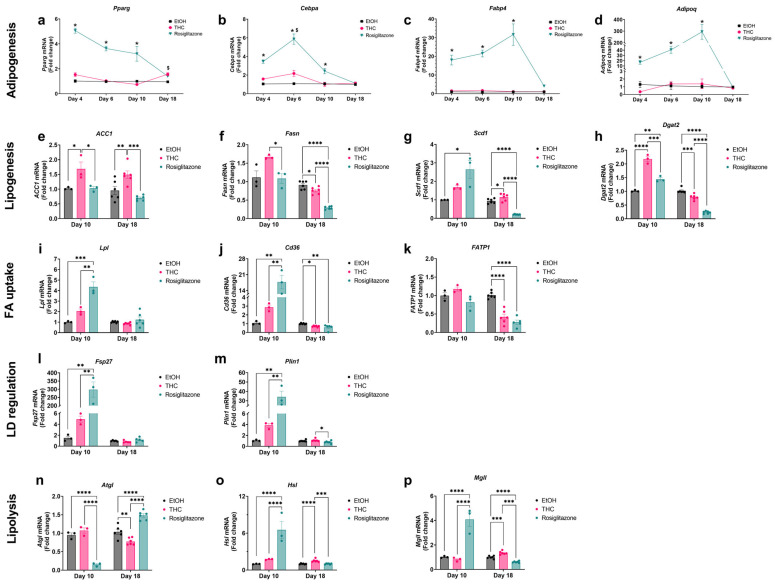
Effects of THC and ROSI on gene expression of adipogenesis and key pathways of lipid metabolism in 3T3-L1 adipocytes. 3T3-L1 preadipocytes were differentiated and treated with vehicle (EtOH, 0.06%), THC (1 µM), or ROSI (30 µM) under FFA-enriched conditions. mRNA expression of key genes was measured by RT-qPCR. Adipogenesis markers (*Pparg*, *Cebpa*, *Fabp4/aP2*, *Adipoq*) were assessed at days 4, 6, 10, and 18 (panels (**a**–**d**), respectively). All remaining genes were analyzed at days 10 and 18 and are grouped by pathway: lipogenic genes (*Acaca*/*ACC*, *Fasn*, *Scd1, Dgat2*; panels (**e**–**h**), respectively), fatty acid uptake genes (*Lpl*, *Cd36*, *Slc27a1*/*FATP1*; panels (**i**–**k**), respectively), lipid droplet-associated genes (*Plin1*, *Cidec*/*Fsp27*; panels (**l**,**m**), respectively), and lipolysis-related genes (*Pnpla2*/*Atgl*, *Lipe*/*Hsl*, *Mgll*; panels (**n**–**p**), respectively). Gene expression values are presented as fold change (ΔΔCt) relative to vehicle-treated controls, normalized to the housekeeping gene *Tbp*. Bars represent mean ± SEM; day 10 includes 3 biological replicates, and day 18 includes 6. For line graphs (panels (**a**–**d**)), statistical significance was determined by two-way ANOVA. Symbols indicate: * Significant difference (*p* < 0.05) for ROSI compared to both vehicle and THC; ^$^ *p* < 0.05 for THC compared to vehicle. For bar graphs (panels (**e**–**p**)), statistical analysis was performed using one-way ANOVA, followed by Tukey’s post hoc test for each gene at each time point. * *p* < 0.05, ** *p* < 0.01, *** *p* < 0.001, **** *p* < 0.0001.

To explore treatment-specific differences in lipid handling, we analyzed gene expression related to adipogenesis, lipogenesis, FA uptake, and LD regulation at key differentiation stages. ROSI robustly increased adipogenic markers (*Pparg*, *Cebpa*, *Fabp4/aP2*, *Adipoq*) through day 10 (Figure 4a–d). By day 18, these markers were similar across groups except for *Pparg*, which was elevated in THC-treated cells (Figure 4a). On day 10, THC increased lipogenic genes (*Fasn*, *Acaca*/*ACC1*, *Dgat2*), while ROSI primarily upregulated *Scd1* (Figure 4e–h). By day 18, THC further increased *ACC1* and *Scd1*, while vehicle controls showed higher *Fasn* and *Dgat2* expression (Figure 4e–h). ROSI elevated FA uptake genes (*Cd36*, *Lpl*) on day 10, aligning with early lipid accumulation (Figure 4i,j). By day 18, vehicle-treated cells exhibited higher *Slc27a1*/*FATP1* and *Cd36* expressions than THC and ROSI (Figure 4j,k). On day 10, ROSI upregulated LD-associated genes (*Plin1*, *Cidec*/*Fsp27*), consistent with increased LD size and maturation (Figure 4l,m). By day 18, THC-treated adipocytes displayed higher *Plin1* levels than ROSI (Figure 4m). Lipolytic gene also varied temporally: ROSI downregulated *Pnpla2*/*Atgl* but elevated expression of *Lipe*/*Hsl* and *Mgll* at day 10; THC mirrored this by day 18, while ROSI reversed its effect (Figure 4n–p). Endocannabinoid system (ECS) gene expression remained unchanged between the THC and vehicle groups, while the *Cnr1* (CB1 receptor) was undetectable on day 10 and reduced in ROSI-treated cells by day 18 (Supplementary Figure S6).

### 2.5. Adipocyte-Hepatocyte Conditioned Medium Model Demonstrates the Functional Consequences of THC-Enhanced FFA Buffering

**Figure 5:**
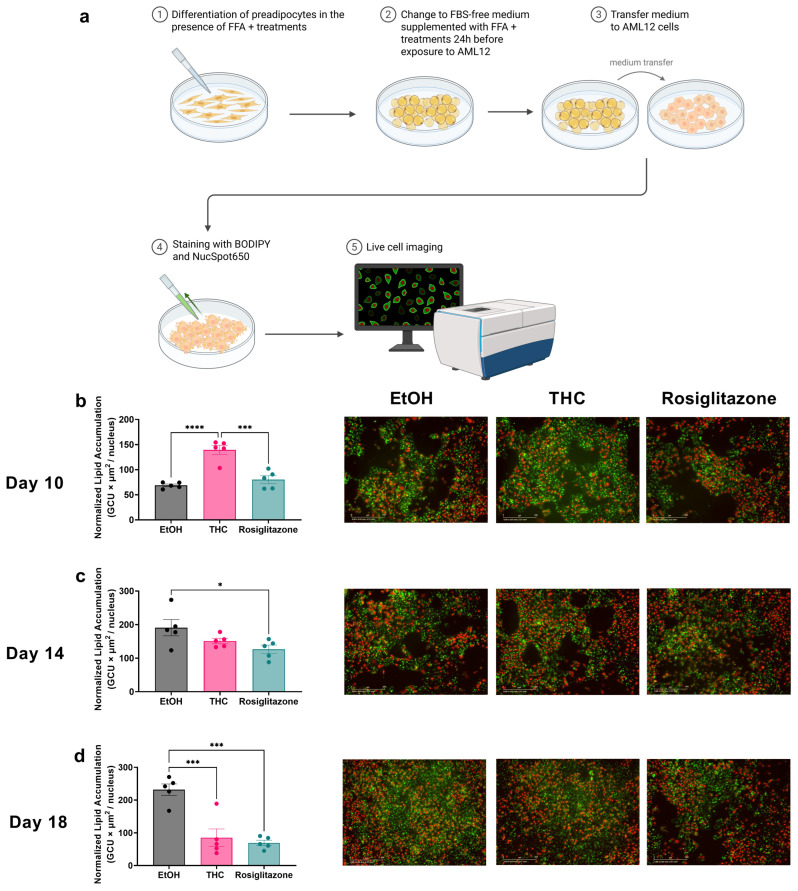
Hepatocyte lipid accumulation following exposure to CM from THC- or ROSI-treated adipocytes at different maturation stages. AML12 hepatocytes were exposed for 24 h to CM collected from 3T3-L1 preadipocytes treated with vehicle (EtOH, 0.06%), THC (1 µM), or ROSI (30 µM) on days 10, 14, or 18 post-induction of differentiation. Lipid accumulation was assessed by BODIPY (green) staining of neutral lipids and normalized to nuclear count using NucSpot650 (red). (**a**) Illustration of the experimental design (**b**–**d**). Quantification of normalized lipid accumulation in hepatocytes following exposure to CM from adipocytes at day 10, day 14, and day 18, respectively. Representative fluorescence images for each treatment condition are shown alongside each corresponding graph. Scale bar 200 µm. Data represent mean ± SEM from (n = 5). Statistical significance was performed using one-way ANOVA followed by Tukey’s post hoc test. * *p* < 0.05, *** *p* < 0.001, **** *p* < 0.0001.

To assess whether adipocyte adaptations influence hepatocyte lipid accumulation, we utilized a CM approach, transferring media from 3T3-L1 adipocytes (treated as described above) to AML12 hepatocytes at days 10, 14, and 18 of differentiation. To maintain FFA/BSA ratio—a critical determinant of hepatocyte lipid uptake and lipotoxicity [12]—adipocytes were switched to FBS-free medium containing the same FFA and treatment concentrations 24 h prior to medium collection (Methods, Figure 5a). Validation experiments confirmed that CM from untreated adipocytes significantly reduced hepatocyte lipid accumulation compared to direct FFA exposure, indicating effective adipocyte buffering (Supplementary Figure S7a–f). When examining the treatment-specific effects, CM from THC-treated adipocytes at day 10 significantly increased hepatocyte lipid accumulation compared to vehicle and ROSI (Figure 5b). By day 14, CM from ROSI-treated adipocytes significantly reduced lipid accumulation relative to vehicle (Figure 5c). By day 18, both THC and ROSI CM reduced hepatocyte lipid accumulation compared to vehicle (Figure 5d). These patterns aligned with NEFA concentrations in corresponding CM samples (Figure 3g–i), supporting a direct link between adipocyte FFA buffering and hepatocyte lipid load. To confirm these effects were adipocyte-mediated, we directly treated AML12 cells with THC, ROSI, or vehicle; no significant changes in lipid accumulation were observed (Supplementary Figure S8).

## 3. Discussion

Adipose tissue expandability is a key determinant of metabolic health, buffering excess lipids to prevent ectopic deposition and reduce MASLD risk [1]. Here, we investigated THC’s role in modulating adipogenesis and FFA handling under obesogenic stress and assessed downstream effects on hepatocyte lipid accumulation using a CM model. Our findings demonstrate that THC enhances adipogenic capacity and improves adipocyte lipid buffering, leading to reduced hepatocyte lipid storage.

While in vivo studies using diet-induced obese models have shown that prolonged THC administration attenuates weight gain, often linked to decreased adiposity and/or smaller adipocytes [10,13,14,15], the direct effects on adipogenesis and adipocyte function under obesogenic conditions remain elusive. Prior in vitro studies lacked physiologically relevant stressors such as excess FFAs [16,17]. We addressed this by mimicking the hyperlipidemic milieu characteristic of obesity, finding that 1 µM THC promoted adipogenesis, reflected by increased lipid accumulation and expression of adipogenic markers. THC also activated human PPARγ, the master regulator of adipogenesis, in a reporter assay, albeit with lower potency than ROSI. This is in line with previous reports identifying THC as a PPARγ activator [18,19]. However, since functional studies were performed in murine adipocytes, further validation of THC’s activation of murine PPARγ is needed to support receptor-specific interpretations [20].

Beyond its role in adipocyte differentiation, PPARγ also improves lipid handling in adipose tissue, with therapeutic implications in metabolic diseases [7]. Though THC primarily acts via the CB1 receptor, the key receptor of the ECS involved in appetite, metabolism, and energy balance [21], growing evidence supports its PPARγ-dependent activity. Such effects have been reported in neuronal, hepatic, and vascular models [18,22,23]. Moreover, THC’s high lipophilicity and preferential accumulation in adipose depots following systemic exposure further support its capacity to directly influence adipocyte function [24]. In support, our previous study in DIO mice showed that chronic THC reduced adipocyte size and accumulated in adipose tissue without altering ECS markers [10]. Here, CB1 expression remained undetectable on day 10 and unchanged on day 18, pointing toward a CB1-independent mechanism. THC-treated preadipocytes also displayed greater proliferation than those treated with ROSI at 48–72 h post-treatment. Though not significant relative to the vehicle, this early proliferative effect—together with moderate PPARγ activation—may contribute to THC’s unique adipogenic profile [25].

Our findings suggest that THC and ROSI modulate lipid storage and FA handling through distinct temporal programs. ROSI enhanced lipid storage and reduced NEFA by day 14, while THC’s effects emerged on day 18. Notably, THC-treated cells showed elevated NEFA at day 10 despite similar lipolysis and lipid content, suggesting incomplete re-esterification—a hallmark of early FFA overload [26,27]. By day 18, lower NEFA and increased storage indicated improved buffering. ROSI’s effects were more progressive, likely due to sustained adipogenic and lipogenic gene activation [28].

Treatment-specific differences in LD architecture also emerged. THC-treated adipocytes consistently displayed a higher number of lipid droplets compared to ROSI, and by day 18, showed a significantly greater number of small LDs than vehicle. In contrast, ROSI promoted larger, more consolidated LDs. These features may reflect distinct lipid turnover dynamics, with THC’s pattern aligning with improved metabolic flexibility and reduced ER stress [29,30].

Both treatments enhanced lipolytic responsiveness. ROSI increased both basal and ISO-stimulated lipolysis by day 14, with further ISO sensitivity by day 18. THC selectively enhanced ISO-stimulated lipolysis at days 10 and 18—a response not seen in vehicle-treated adipocytes—suggesting regained catecholamine sensitivity under lipotoxic conditions [30].

Transcriptomic data support THC’s temporally dynamic effects. On day 10, THC upregulated lipogenic enzymes (*Acaca*, *Fasn*, *Dgat2*), alongside trends toward increased expression of FA uptake genes (*Lpl*, *Cd3*) and LD-associated proteins (*Fsp27*, *Plin1*), indicating early anabolic adaptation. However, NEFA remained high, consistent with re-esterification inefficiency [31,32]. By day 18, expression of *Fasn*, *Dgat2*, and *Cd36* declined, likely reflecting feedback inhibition of lipid influx as storage capacity was reached [33]. Meanwhile, *Acaca* and *Scd1* remained elevated, and *Pparg* was upregulated, suggesting active lipid turnover and unsaturated FA synthesis [27].

These temporal shifts suggest that prolonged THC exposure initially challenges FFA sequestration—possibly due to lagging re-esterification efficiency—but ultimately promotes a remodeled, more effective lipid-handling state. This pattern parallels reports linking adolescent THC exposure to altered adipose function and aligns with epidemiological data associating chronic cannabis use with improved insulin sensitivity and visceral fat regulation [10,13,14].

Using a CM approach, we demonstrated that NEFA levels in adipocyte media closely reflected lipid accumulation in hepatocytes, supporting the idea that adipocyte buffering dictates hepatic FFA exposure [34]. Notably, CM from THC- and ROSI-treated adipocytes at day 18 reduced hepatocyte lipid storage, even when NEFA differences were modest- suggesting possible involvement of other adipocyte-derived factors, a possibility beyond this study’s scope.

Despite its conceptual utility, the CM model lacks physiological complexity. In vivo, hepatocytes acquire lipids mainly from chylomicron remnants and LDL via receptor-mediated uptake, and from FFA released by lipoprotein lipase action on circulating lipoproteins [35]. Our in vitro conditions also lack dynamic flux and systemic hormonal cues present in human metabolic diseases [36]. Nevertheless, our findings highlight the central role of adipocyte FFA buffering in modulating hepatic lipid exposure and underscore the value of the CM model for dissecting adipose-liver metabolic interactions.

## 4. Materials and Methods

### 4.1. 3T3-L1 Cell Culture and Differentiation

The 3T3-L1 mouse preadipocytes (ATCC CL-173) were cultured in DMEM (Cat# D5796, Sigma-Aldrich, St. Louis, MO, USA) supplemented with 10% calf serum (Cat# C8056, Sigma, St. Louis, MO, USA), 1 mM sodium pyruvate, and 1% penicillin–streptomycin (Cat# D910-100M Lifegene, Mevo Horon, Israel) at 37 °C and 5% CO_2_. For differentiation, cells were seeded in 96-well plates (3.5 × 10^4^ cells/cm^2^) in a volume of 300 µL per well, and two days post-confluence (day 0), switched to differentiation medium containing 10% fetal bovine serum (Cat# S1400, Sigma-Aldrich, St. Louis, MO, USA), 0.5 mM IBMX (Cat# 410957, Sigma-Aldrich, St. Louis, MO, USA), 1 μM dexamethasone (Cat#D4902, Sigma-Aldrich, St. Louis, MO, USA) and 10 μg/mL insulin (Cat# I9278, Sigma-Aldrich) following standard protocols for 3T3-L1 adipocyte differentiation [37]. After 48 h, cells were maintained in the same medium with 10% FBS and insulin only, refreshed every 48 h. From day 0, cells were treated with vehicle (ethanol [EtOH], 0.06% final concentration, 3 µL), THC (1 μM, 3 µL; BOL Pharma, Industrial Area Revadim, Israel), or ROSI (30 mM, 0.3 µL; (Cat# R2408 Sigma-Aldrich, St. Louis, MO, USA), which served as a positive control. The THC concentration was selected based on our dose–response experiments and supported by prior in vitro studies showing cellular effects and tolerability at similar ranges [23,38]. All treatments included 0.5 mM of an FFA mixture (oleate/palmitate, 2:1 molar ratio; Cat# O5504 and Cat# P9767, Sigma-Aldrich, St. Louis, MO, USA), conjugated to fatty-acid-free BSA (Cat# 126579, Sigma-Aldrich, St. Louis, MO, USA), to mimic lipid-rich conditions (FFA/BSA ≈ 5.3:1) as described previously [12].

### 4.2. Cell Viability Assessment (MTT and Propidium Iodide Staining)

Cell viability was assessed using MTT and propidium iodide (PI) assays. For MTT (Cat# 537060, Sigma-Aldrich, St. Louis, MO, USA), 0.5 mg/mL solution was added to treated cells in 96-well plates at days 0, 2, 4, and 6, and incubated for 3 h at 37 °C. Cells were exposed to THC at 10 nM, 1 µM, and 10 µM to evaluate potential cytotoxicity across a concentration range. Formazan was solubilized in DMSO, and absorbance was read at 570 nm (Tecan Infinite 200 PRO, Männedorf, Switzerland). To assess membrane integrity and cell death, PI staining was performed following differentiation induction. For PI staining (Cat# 25535-16-4, Sigma-Aldrich), 5 μg/mL PI in complete medium was added 1:1 to wells post-differentiation induction (final 2.5 μg/mL). vehicle-treated cells served as negative controls. Phase-contrast and red fluorescence imaging were performed at 24 and 48 h using the IncuCyte live-cell system (Sartorius, Göttingen, Germany), and PI-positive cells were quantified based on red fluorescence object count using IncuCyte software (version 2020B).

### 4.3. Cell Proliferation Assay

Preadipocyte proliferation was monitored using the IncuCyte live-cell imaging system (Sartorius) under standard conditions (37 °C, 5% CO_2_). 3T3-L1 cells were seeded in 96-well plates (2500 cells/well) and treated 24 h later with vehicle (EtOH, 0.06%), THC (1 µM), or ROSI (30 µM). Phase-contrast images were captured every 12 h for 72 h using a 10× objective. Cell counts were quantified with IncuCyte software (default segmentation for 3T3s), normalized per image field, and exported for analysis.

### 4.4. Oil Red O Staining and THC Dose–Response Assay

Lipid accumulation was assessed by Oil Red O (ORO; Cat# O0625, Sigma-Aldrich, St. Louis, MO, USA) staining as previously described [13]. Briefly, cells were fixed in 10% formalin, stained with 0.3% filtered ORO solution, and washed. Lipid-bound dye was eluted using isopropanol, and absorbance was measured at 492 nm (Tecan Infinite 200 PRO). To determine the optimal concentration of THC for promoting adipogenesis, 3T3-L1 preadipocytes were differentiated with 10 nM, 1 μM, or 10 μM THC from day 0; EtOH-treated and undifferentiated cells served as vehicle and negative controls, respectively. Lipid content was quantified on days 4 and 6. Based on this analysis, 1 μM THC was used in all subsequent assays.

### 4.5. Live Cell Imaging and Staining

The 3T3-L1 adipocytes were stained with BODIPY™ 493/503 (0.3 µM; Cat# D3922 Thermo Fisher Scientific, Carlsbad, CA, USA) and NucSpot650 (1:1000; Cat# BTM-400820, Biotium, Fremont, CA, USA) in serum-free medium and imaged using the IncuCyte live-cell imaging system (Sartorius), following manufacturer protocols.

#### 4.5.1. LD Dynamics and Lipogenic Capacity

To track LD dynamics, 3T3-L1 adipocytes were stained with BODIPY on day 10 of differentiation and imaged every 24 h until day 16. At each time point, the medium was replaced with BODIPY staining solution diluted 2:1 with fresh adipocyte maintenance medium. Images were acquired using the IncuCyte live-cell imaging system (Sartorius). LDs were identified by applying a fixed fluorescence intensity threshold across all conditions to minimize background signal. Object segmentation was performed with the IncuCyte analysis software (version 2020B), which quantified: (i) LD size, determined from segmented droplet area (μm^2^); (ii) LD count, recorded as the number of discrete objects per image; and (iii) integrated fluorescence intensity, calculated as the sum of BODIPY signal across all segmented droplets within each image. Data were averaged across replicate images for each condition and time point.

#### 4.5.2. Single Time Point Lipid Accumulation

Adipocytes were stained with BODIPY and NucSpot650 at days 10, 14, and 18, then imaged by IncuCyte. Lipid accumulation was calculated as integrated BODIPY fluorescence normalized to nuclear count, yielding lipid per cell.

#### 4.5.3. Lipid Droplet Size Classification

LDs were classified as small (≤25 μm^2^), medium, or large (≥125 μm^2^) using percentile thresholds (25th/75th percentiles) derived from area distributions obtained from IncuCyte-generated histograms of BODIPY-stained images. For each condition, the proportion of LDs falling into each size category (small, medium, and large) was calculated relative to the total number of droplets detected. This approach enabled consistent cross-condition comparisons and captured treatment-induced shifts in LD morphology.

### 4.6. Conditioned Medium Treatment of AML12 Cells

AML12 hepatocytes (ATCC, CRL-2254) were seeded in 96-well plates at 2 × 10^5^ cells/cm^2^. After reaching confluence, culture medium was replaced with conditioned medium (CM) collected from 3T3-L1 adipocytes at days 10, 14, or 18. To ensure consistent BSA/FFA ratios in the CM, 3T3-L1 adipocyte cultures were switched to FBS-free medium 24 h prior to CM collection, while treatment conditions remained unchanged. AML12 cells were incubated with CM for 24 h, then stained with BODIPY and NucSpot650 to quantify normalized lipid accumulation.

### 4.7. PPARγ Luciferase Reporter Assay

PPARγ activation was assessed using luciferase reporter assay (Cat# IB00111, INDIGO Biosciences, State College, PA, USA) following the manufacturer’s protocol. Briefly, reporter cells were plated in 96-well plates and treated with increasing concentrations of THC or ROSI. After 24 h at 37 °C in a humidified 5% CO_2_ incubator, luciferase activity was measured by luminometry. Luminescence values were log-transformed to generate dose–response curves and calculate EC_50_ values.

### 4.8. Lipolysis Assay

Lipolysis was measured in differentiated 3T3-L1 adipocytes after 24 h in insulin-free medium. Cells were washed with PBS and incubated for 3 h in phenol-red-free DMEM with 5% fatty-acid-free BSA, with or without 10 μM isoproterenol (Sigma-Aldrich). Conditioned media were collected, and glycerol release was quantified colorimetrically (Cat# MAK117, Sigma-Aldrich) at 540 nm (Tecan Infinite 200 PRO). Values were normalized to total protein by BCA assay (Cat# 23227, Thermo Fisher) and expressed as nmol glycerol per μg protein.

### 4.9. Non-Esterified Fatty Acids (NEFA) Quantification

NEFA levels were measured in culture supernatants using a colorimetric assay kit (Cat# E-BC-K728-M, Elabscience, Houston, TX, USA) per the manufacturer’s protocol. Samples were incubated with assay reagents, and absorbance was read at 550 nm (Tecan Infinite 200 PRO). Concentrations (μmol/L) were calculated from a standard curve. All samples were run in duplicate with background subtraction.

### 4.10. RNA Isolation and Quantitative RT-PCR

Total RNA was extracted using the NucleoSpin^®^ RNA kit (Cat#740955, Macherey-Nagel, Düren, Germany) and quantified via NanoDrop (Nanodrop 2000 Thermo Fisher Scientific, Carlsbad, CA, USA). cDNA was synthesized from 1 µg RNA (Cat# 95047-500, Quanta BioSciences, Beverly, MA, USA), and qPCR was performed using SYBR Green master mix (Cat# AB-A46113, Thermo Fisher) on a QuantStudio™ system (Applied Biosystems, Foster City, CA, USA). Gene expression levels were normalized to TATA-binding protein (Tbp) and calculated usingthe 2^−ΔΔCt^ method. Primer sequences are listed in Supplementary Table S1.

### 4.11. Statistical Analysis

Statistical analyses were performed using GraphPad Prism version 9 (GraphPad Software, San Diego, CA, USA). Data are presented as mean ± SEM. Comparisons between groups at each time point were conducted using one-way ANOVA followed by Tukey’s post hoc test, or two-way ANOVA with Tukey’s correction for multiple comparisons as appropriate. Statistical significance was defined as *p* <  0.05.

## 5. Conclusions

Collectively, these findings provide the first evidence that THC promotes a metabolically adaptive adipocyte phenotype under obesogenic conditions, balancing lipid storage and mobilization to limit ectopic lipid deposition. Importantly, our results highlight that both the timing and context of THC exposure are critical for these beneficial effects, underscoring the need to consider the metabolic state and exposure.

## Data Availability

The raw data supporting the conclusions of this article will be made available by the authors on request.

## Supplementary Materials

The following supporting information can be downloaded at https://www.mdpi.com/article/10.3390/ijms26188860/s1.
