# Supplementary material for: Distinct Adipocyte Responses to Δ9-Tetrahydrocannabinol (THC) Exposure Govern Hepatic Lipid Accumulation in an Obesogenic Setting
Source: Int J Mol Sci. 2025 Sep 11;26(18):8860. doi: 10.3390/ijms26188860 (PMC12469841; doi:10.3390/ijms26188860)
Supplement: Supplementary file 1 [file ijms-26-08860-s001.zip › ijms-3841198-supplementary.pdf]

International Journal of
Molecular Sciences

MDPI

Supplementary

Distinct Adipocyte Responses to Δ-9-tetrahydrocannabinol (THC) Exposure Govern Hepatic Lipid Accumulation in an Obesogenic Setting

Adi Eitan, Ofer Gover† and Betty Schwartz *,†

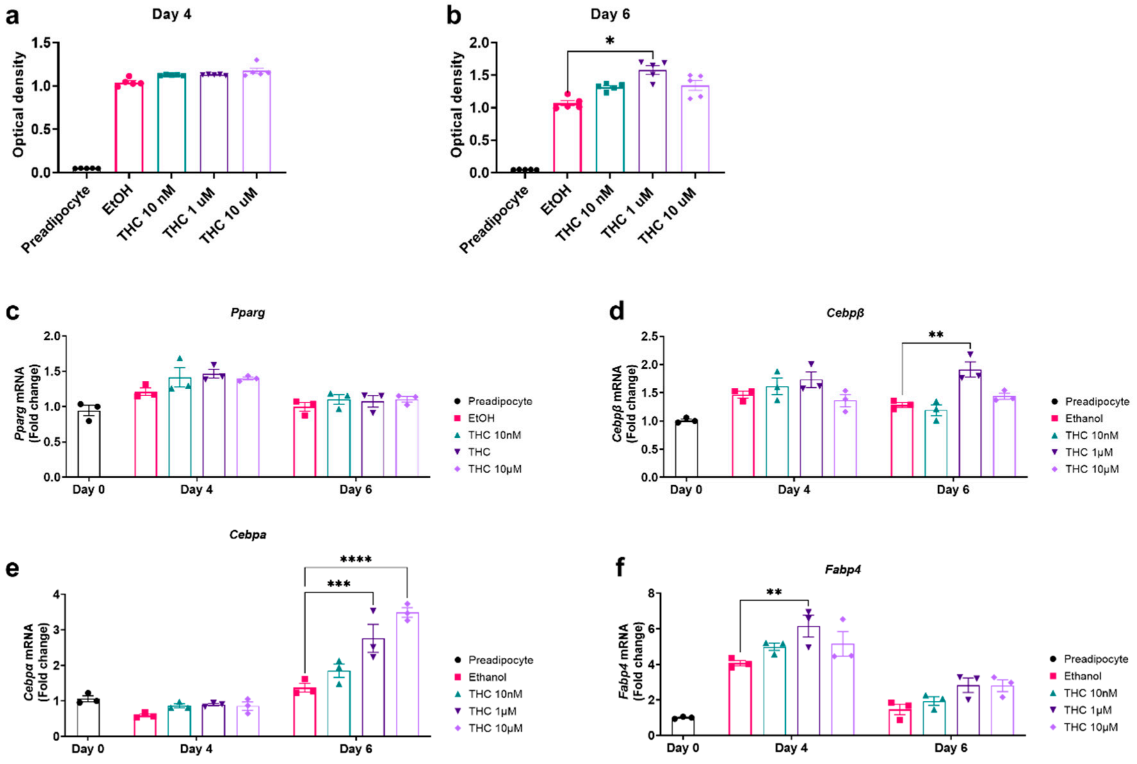

Figure S1. Dose-dependent effect of THC on 3T3-L1 adipocyte differentiation and adipogenic gene expression. 3T3-L1 preadipocytes were differentiated under FFA-enriched conditions in the presence of vehicle (EtOH, 0.06%) or increasing concentrations of THC (10 nM, 1 μM, 10 μM), administered at the initiation of differentiation (day 0). Quantification of Oil Red O (ORO) staining by optical density on day 4 (a) and day 6 (b) of differentiation. Relative mRNA expression (fold change vs. day 0) of key adipogenic transcription factors and markers: *Pparg* (c), *Cebpβ* (d), *Cebpα* (e), and *Fabp4* (f) at indicated time points and treatments. Data are presented as mean ± SEM (n=3). ORO data was analysed by one-way ANOVA with Tukey's post hoc test. Gene expression data were analyzed by two-way ANOVA followed by Tukey's post hoc test for comparisons within each time point. *$p < 0.05$; **$p < 0.01$; ***$p < 0.001$; ****$p < 0.0001$.

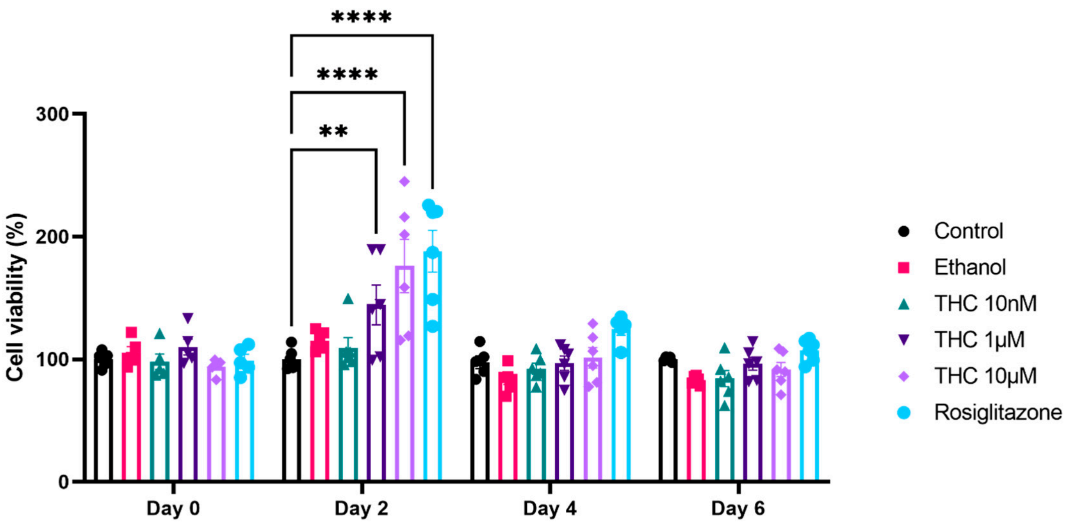

Figure S2. MTT assay assessing cell viability during adipocyte differentiation under FFA exposure and various treatments. 3T3-L1 preadipocytes were treated with free fatty acids (FFA) in combination with either control medium, vehicle (EtOH, 0.06%) , THC (10 nM, 1 μM, or 10 μM), or Rosiglitazone (ROSI, 30 μM). Cell viability was measured on days 0, 2, 4, and 6 of differentiation using the MTT assay. Results are expressed as percent viability relative to untreated control cells. Data are presented as mean ± SEM (n=5). Two-way ANOVA determined statistical significance with Tukey's post hoc test: **p < 0.01; ****p < 0.0001.

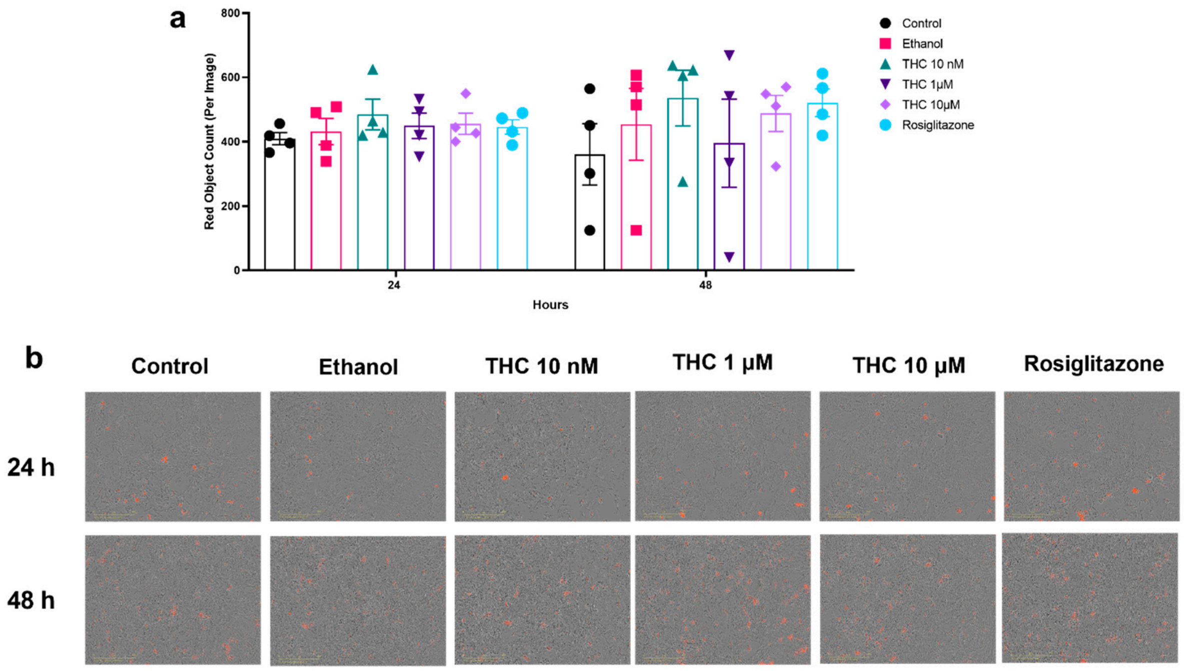

Figure S3. Propidium iodide (PI) staining of 3T3-L1 cells under FFA-rich conditions 24 and 48 hours after induction of differentiation. Cells were treated with vehicle (EtOH, 0.06%), THC (10 nM, 1 μM, or 10 μM), or Rosiglitazone (ROSI, 30 μM), and PI staining was performed using the IncuCyte live-cell imaging system. The graph (a) shows quantification of red object count per image, representing PI-positive cells with compromised membrane integrity (i.e., dead or dying cells). Representative images acquired at 10× magnification are shown in (b). Data are presented as mean ± SEM (n = 4).

Statistical analysis was performed using two-way ANOVA; no significant differences were observed between treatment groups.

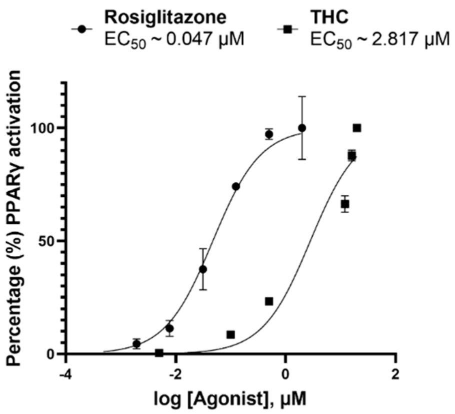

Figure S4. THC activates PPARγ with lower potency compared to rosiglitazone as determined by luciferase reporter assay. Reporter cells were treated with increasing concentrations of rosiglitazone (circles) or THC (squares) for 24 hours. Transcriptional activation was measured using the Indigo Biosciences luciferase reporter assay kit. Data are presented as a percentage of maximal PPARγ activation relative to rosiglitazone (100%) and plotted against the log concentration of agonist. Values represent mean ± SEM (n=3).

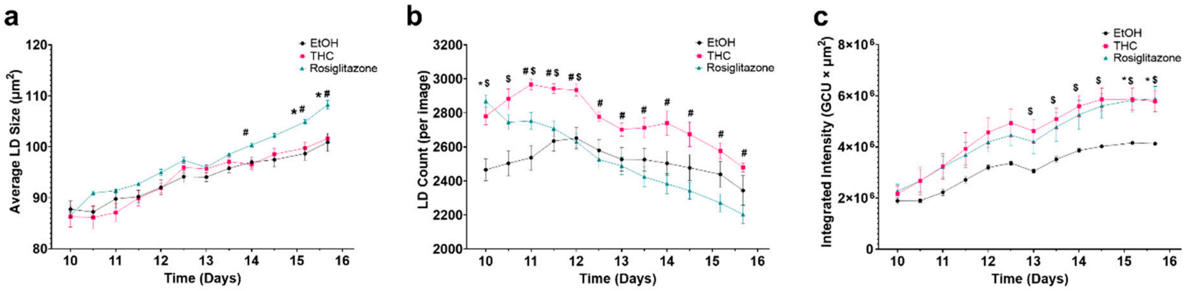

Figure S5. THC and rosiglitazone differentially modulate lipid droplet (LD) dynamics and lipid accumulation in mature adipocytes. 3T3-L1 preadipocytes were differentiated and treated with vehicle (EtOH, 0.06%), THC (1 μM), or Rosiglitazone (30 μM) under FFA-enriched conditions, stained with BODIPY, and monitored from day 10 to day 16 post-induction of differentiation. (a) average LD size, (b) LD count per image, and (c) integrated intensity were measured every 24 hours using IncuCyte live cell imaging system. Data are presented as mean ± SEM (n=5). # indicates $p < 0.05$ THC vs. Rosiglitazone; $ indicates $p < 0.05$ THC vs. Vehicle control; * indicates $p < 0.05$ vehicle control vs. Rosiglitazone as determined by two-way ANOVA with Tukey's post hoc test for comparisons within time points.

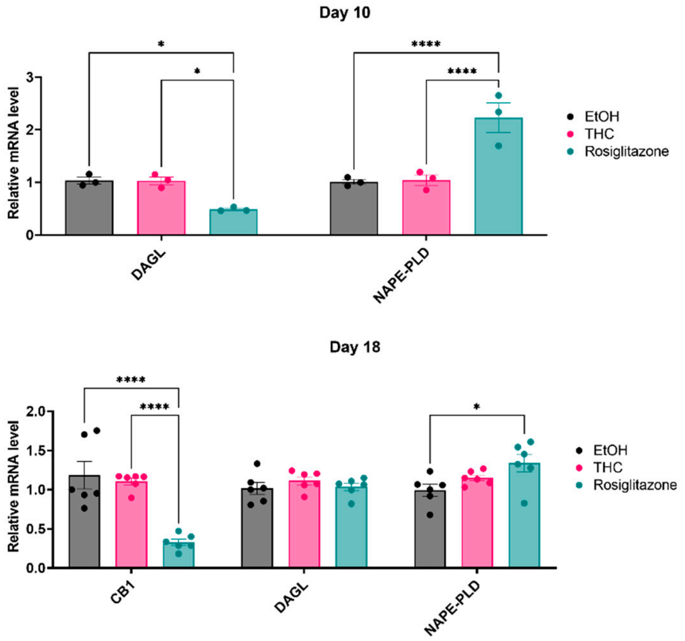

Figure S6. Effects of THC and Rosiglitazone on gene expression of ECS. 3T3 adipocytes were differentiated and treated with vehicle (EtOH), THC, or Rosiglitazone (ROSI) under FFA-enriched conditions. mRNA expression of key genes of ECS was measured by RT-qPCR at day 10 and 18 of differentiation. Gene expression values are presented as fold change (ΔΔCt) relative to vehicle-treated controls, normalized to the housekeeping gene TBP. Bars represent mean ± SEM; day 10 includes 3 biological replicates, and day 18 includes 6. Statistical analysis was performed using two-way ANOVA. $^{*}p < 0.05$, $^{****}p < 0.0001$. .

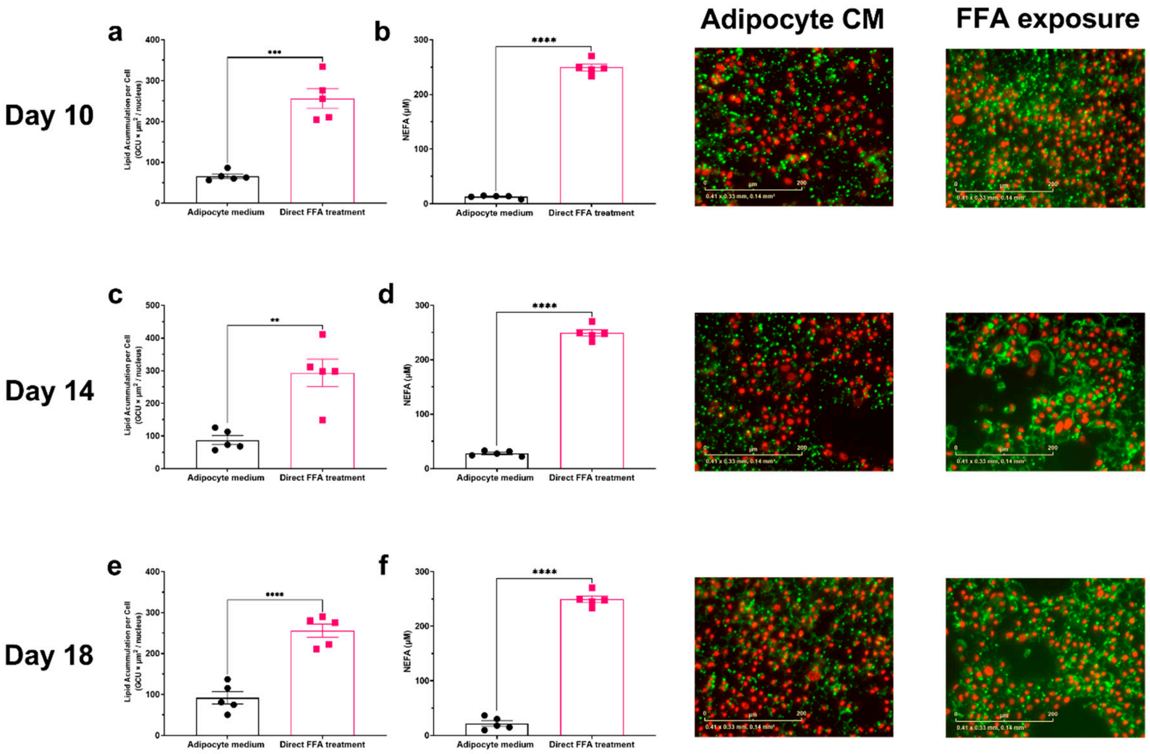

Figure S7. Adipocyte-conditioned medium reduces free fatty acid (FFA) availability and limits lipid accumulation in hepatocytes. AML12 hepatocytes were exposed for 24 hours to either FFA-supplemented medium (0.5 mM oleate: palmitate, 2:1 molar ratio) or conditioned medium (CM) collected

from 3T3-L1 adipocytes at days 10, 14, or 18 of differentiation.
(a, c, e) Lipid accumulation in hepatocytes was assessed by BODIPY 493/503 (green) staining and normalized to nuclear count, based on red object detection using NucSpot650 (red). Data are presented as normalized fluorescence units. (b, d, f) NEFA levels were measured from the same media used to treat the hepatocytes, reflecting residual extracellular fatty acid levels. Representative IncuCyte images for each treatment condition are shown to the right of each graph pair scale bar 200 µm. Data are presented as mean ± SEM (n = 5). Statistical significance was determined using Welch's t-test. **$p < 0.01$, ***$p < 0.001$, ****$p < 0.0001$.

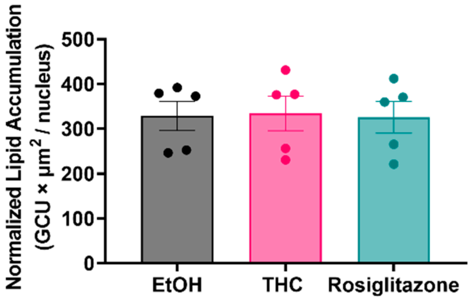

Figure S8. Direct exposure of hepatocytes to THC or Rosiglitazone does not account for the lipid-lowering effects observed with adipocyte-conditioned media. AML12 hepatocytes were treated for 24 hours with THC, Rosiglitazone (ROSI), or vehicle (ethanol) in the presence of free fatty acids (0.5 mM oleate: palmitate, 2:1 molar ratio). Lipid accumulation was assessed by BODIPY 493/503 staining and normalized to nuclear count based on NucSpot650 red object detection. Data are presented as normalized fluorescence units (mean ± SEM, n = 5). Statistical analysis was performed using one-way ANOVA; no significant differences were observed between treatment groups.

Table S1. Primer list.

**Gene**	**Forward primer** / **Reverse primer**	Accession number	Slope	Efficiency (%)
Pparg	ATGTCTCACAATGCCATCAGGT	NM_011146.3	3.26	102
	GAGATCTCCGCCAACAGCTT			
Cebpβ	ATCCGGATCAAACGTGGCT	NM_009883	3.3	101
	AACCCCGCAGGAACATCTTT			
Cebpα	AGTACCGGGTACGGCGGGAAC	NM_007678.4	3.34	99
	GCGTGTCCAGTTCACGGCTCA			
Fabp4	TGGGATGGAAAGTCGACCAC	NM_024406.3	3.36	98.5
	ATCCAGGCCTCTTCCTTTGG			
Adipoq	GACACCAAAAGGGCTCAGGA	NM_009605	3.42	95.7
	ACGTCATCTTCGGCATGACT			
Fasn	GTGATAGCCGGTATGTCGGG	NM_007988	3.38	97.4
	TAGAGCCCAGCCTTCCATCT			
ACC1	TCCACGAAAAGAGCTGACCT	NM_133360.2	3.2	107.9
	ACTAAGGATGCTCCCCACCT			
Scd1	CCAAGCTGGAGTACGTCTGG	NM_009127.4	3.3	98
	CAGAGCGCTGGTCATGTAGT			
Cd36	GCAAAACGACTGCAGGTCAA	NM_007643	3.5	92.9
	GGCCATCTCTACCATGCCAA			
Lpl	TCGTCATCGAGAGGATCCGA	NM_008509.2	3.5	91
	TGTTTGTCCAGTGTCAGCCA			

FATP1	CGCCGATGTGCTCTATGACT<br>ACACAGTCATCCCAGAAGCG	NM_001357182.1	3.1	111
Plin1	GACAAGGAGTCAGCCCCTTC<br>CTCACAAGGCTTGGTTTGGC	NM_001113471.1	3.63	88.3
Fsp27	CCATCAGAACAGCGCAAGAAG<br>AGAGGGTTGCCTTCACGTTC	NM_001372264.1	3.2	102
Atgl	GACAGCTCCACCAACATCCA<br>GCAAAGGGTTGGGTTGGTTC	NM_001163689.1	3.4	98
Hsl	CACAAAGGCTGCTTCTACGG<br>GGAGAGAGTCTGCAGGAACG	NM_010719.2	3.35	92.8
Mgll	ATATGACCTTGGGGCGCATT<br>AGCAGGAATGGCAGTGTCAG	NM_001166251.1	3.2	105
Dgat2	GCCATGGAGCTGATCTGGTT<br>CCCAGGAACCCTCCTCAAAG	NM_026384.3	3.5	91.2
Tbp	CCCTATCACTCCTGCCACAC-CAGC<br>GTG-CAATGGTCTTTAGGTCAAGTTTACAGCC	NM_013684	3.4	95
